# ZNF24 regulates the progression of KRAS mutant lung adenocarcinoma by promoting SLC7A5 translation

**DOI:** 10.3389/fonc.2022.1043177

**Published:** 2022-11-23

**Authors:** Daqi Jia, Leilei Li, Peng Wang, Qiang Feng, Xinyan Pan, Peng Lin, Shuling Song, Lilin Yang, Julun Yang

**Affiliations:** ^1^ Department of Pathology, Kunming Medical University, Kunming, Yunnan, China; ^2^ Department of Pathology, 920^th^ Hospital of the Joint Logistics Support Force of PLA, Kunming, Yunnan, China

**Keywords:** KRAS mutation, lung adenocarcinoma, SLC7A5, ZNF24, protein interaction

## Abstract

**Background:**

Clinical treatment of RAS mutant cancers is challenging because of the complexity of the Ras signaling pathway. SLC7A5 is a newly discovered downstream gene of the Ras signaling pathway, but the regulatory mechanism is unclear. We aimed to explore the molecular mechanism and role in KRAS mutant lung adenocarcinoma progression.

**Methods:**

Key gene that regulated SLC7A5 in KRAS mutant lung adenocarcinoma was screened by RNA sequencing and bioinformatics analysis. The effect of this gene on the expression of SLC7A5 was studied by RNAi. The regulatory mechanism between the two genes was investigated by immunofluorescence, CoIP, pulldown and yeast two-hybrid assays. The location of the two genes was determined by inhibiting Ras and the downstream pathways PI3K-AKT and MEK-ERK. By *in vivo* and *in vitro* experiments, the effects of the key gene on the biological functions of KRAS mutant lung adenocarcinoma were explored.

**Results:**

We found a novel gene, ZNF24, which upregulated SLC7A5 protein expression rather than mRNA expression in KRAS mutant lung adenocarcinoma. Endogenous protein interactions occurred between ZNF24 and SLC7A5. Ras inhibition reduced the expression of ZNF24 and SLC7A5. ZNF24 and SLC7A5 are located downstream of the MEK-ERK and PI3K-AKT pathways. *In vivo* and *in vitro* functional experiments confirmed that the ZNF24-SLC7A5 signaling axis promoted the proliferation, invasion and migration of KRAS mutant lung adenocarcinoma.

**Conclusions:**

ZNF24 promoted the growth of KRAS mutant lung adenocarcinoma by upregulating SLC7A5 protein expression, which suggested that ZNF24 is a new biomarker of KRAS mutant tumors and could be a new potential therapeutic target for Ras-driven tumors.

## Background

Lung adenocarcinoma (LUAD) is one of the most common pathological types of lung cancer and has a complex mechanism, strong invasion and poor prognosis (1–3). Approximately 30% of LUAD patients develop Kirsten rat sarcoma viral oncogene (KRAS) mutations. In addition, patients with LUAD harboring KRAS mutations have worse clinical treatment efficacy, with a worldwide 5-year survival rate of 15% (4, 5). Mutated KRAS affects cell signal transduction by binding with the GTP and becoming activated, activating the downstream tumor signaling pathway, significantly improving cell proliferation ability and influencing immune targeting (6, 7). However, due to the unique location of the Ras protein in the inner membrane, the research and development of KRAS-targeted drugs are very difficult, and the clinical treatment of KRAS mutant LUAD is still challenging (8). Therefore, screening and identifying new downstream markers of the Ras signaling pathway and elucidating their mechanisms are of great theoretical and practical significance for the diagnosis and treatment of KRAS mutant LUAD.

We discovered that the mRNA expression levels of SLC7A5 and ZNF24 genes in KRAS mutant LUAD were significantly increased by transcriptome sequencing combined with online databases, and their expression levels were positively correlated. The purpose of our study was to reveal the mechanism by which KRAS activation increases ZNF24 and SLC7A5 expression. The main function of solute carrier family 7 member 5 (SLC7A5) is to help specific amino acids pass through cell membranes, provide nutrition for tumor cells, and participate in related metabolic pathways (9). Recently, many studies have proven that SLC7A5 expression in adjacent tissues is lower than that in cancer tissues and is closely related to the growth and proliferation of tumor cells (10). SLC7A5 has been reported to be expressed at abnormally high levels in esophageal cancer, hepatocellular cancer, breast cancer, colon cancer and other cancers, and it could be used as a prognostic factor (11). Surprisingly, in 2021, an article in Nature Genetics noted that KRAS mutation in colorectal cancer can induce increased expression of SLC7A5 (12), which further indicates that high expression of SLC7A5 is closely related to KRAS mutation. These results indicated that SLC7A5 could be a potential target for studying KRAS mutant LUAD. ZNF24, also known as ZNF191, is a SCAN subfamily member of Krüppel-like zinc finger transcription factors. As a pleiotropic factor, it is expressed at abnormally high levels in prostate cancer, liver cancer and other cancers and is correlated with malignant proliferation, tumor volume, Gleason score, pathological grade and metastasis of tumor cells (13, 14). However, it has not been reported whether KRAS mutation induces upregulation of ZNF24 and whether ZNF24 regulates SLC7A5 expression. Clarification of the mechanism of ZNF24 is very important for elucidating the development of KRAS mutant LUAD.

In this study, ZNF24 and SLC7A5 were identified as differentially expressed in KRAS mutant LUAD, and the specific regulatory mechanisms were further studied, revealing the biological behavior changes of KRAS mutant LUAD under the regulation of the ZNF24-SLC7A5 signaling axis and providing a theoretical basis for exploring new therapeutic targets. Our results support the potential application of this signaling axis in the diagnosis, prognosis prediction, and treatment of KRAS mutant LUAD.

## Methods

### Patients and samples

From 2012 to 2022, thirty-six LUAD tissues and matched paracancerous specimens were obtained from patients undergoing LUAD resection in 920^th^ Hospital of the Joint Logistics Support Force of PLA. Preoperative ultrasound, CT or MRI was used to determine the extent of the lesion, and the inclusion criteria were a clear diagnosis of LUAD by pathology and a clear KRAS status by genetic testing. The patients did not receive radiotherapy chemotherapy or targeted therapy before surgery. The process of sample collection was approved by the ethics committee of 920^th^ Hospital of the Joint Logistics Support Force of PLA. The clinical characteristics of the KRAS-mutated LUAD patients are shown in **Table 1**.

**Table 1:** Clinical features of KRAS-mutated LUAD patients.

Features	Variables	No. (%)
Age	**<** 60	19 (53)
	**≥** 60	17 (47)
Gender	Male	20 (56)
	Female	16 (44)
Pathological stage	Early (T1-T2)	15 (42)
	Advanced (T3-T4)	21 (58)
Lymphatic metastasis	Yes	10 (28)
	No	26 (72)
KRAS Status	KRAS Wild	28 (78)
	KRAS Mutation	8 (22)

### Data from the TCGA cohort

Corresponding clinical information of LUAD patients and RNA-seq data were obtained from the Gene Expression Omnibus (https://www.ncbi.lm.nih.gov/geo/). According to the median ZNF24 and SLC7A5 mRNA expression of each sample, patients were divided into the low or high expression group. In this study, the GSE72094 dataset was downloaded from the GEO database, which contained the full transcriptomic data of 288 wild-type KRAS cases and 154 mutant KRAS cases. After downloading the data, R software was used for data processing, including standardization and differential expression value calculation, and log fold-change (log FC) and Student’s t-test were used for differential expression analysis and screening.

### Immunohistochemical staining and tissue microarray analysis

Tissue sections were first incubated for 2 hours at 68°C; dewaxed with xylene, anhydrous ethanol, a gradient ethanol series and distilled water; and then boiled in acid buffer (pH of 6.0) for 2 min at a high temperature and pressure. Then, endogenous peroxidase activity was blocked with 0.3% hydrogen peroxide. The sections were incubated with 5% normal goat serum for 30 min, and then ZNF24 antibody (11219-1-AP, Proteintech) and SLC7A5 antibody (28670-1-AP, Proteintech) were diluted at a ratio of 1:200 and incubated at 4 °C overnight. Goat anti-mouse IgG-HRP or Goat anti-rabbit IgG-HRP (ready-to-use, DAKO) was incubated at 37°C for 1h and then stained with DAB for 30s-60s.

### Cell culture and lentivirus transfection

All cell lines used in this study were purchased from The Center for Molecular and Cellular Sciences (Shanghai, China). They were incubated at 37°C and humidified with 5% CO2 in the presence of 10% fetal bovine serum (VivaCell), 100 μg/ml streptomycin and 100 units/ml penicillin.

H358 and A549 cell lines were transfected with lentiviruses carrying knockdown (or overexpression) constructs for the ZNF24 and SLC7A5 genes respectively, stably increasing or decreasing the expression of ZNF24 and SLC7A5. Lentiviruses were purchased from Genechem (Shanghai, China). H358 and A549 cells (2×10^4^) were grown in 6-well plates and transfected using lentiviruses (MOI = 10, 20 μL) when the cells were 70% confluent. After 6 hours, the RPMI 1640 medium containing 10% FBS was changed, and the culture was continued for 48 h. The cells were treated with a mixture containing 2 μg/mL puromycin in RPMI 1640 medium (3 - 5 d). The effect of knockdown and overexpression was detected by qPCR and WB, respectively (as shown below).

### RNA extraction, reverse transcription, and real-time fluorescent quantitative PCR assay

Total RNA was isolated from tissues or cells according to the instructions of the RNA extraction kit (Promega, LS1040, Shanghai, China). Then, cDNA was synthesized using a fixed one-step RT‐PCR kit (Promega, A6120, Shanghai, China). qRT‐PCR experiments were completed using the SYBR Green Super Mix system (Tsingke Biotechnology, TSE201, Beijing, China). The experiment was repeated in at least 3 separate experiments, and the changes in gene expression were evaluated by the 2^−ΔΔCT^ method. The primer sequences used for qRT‐PCR were as follows: human SLC7A5:F:5′-CCTGCCTGTGTTCTTCAT-3′andR:5′-GCTGAGGATGATGGTGAA-3′;human ZNF24:F:5′-TGGAGCACTAGCTCCAAAGC-3′andR:5′-CGTCGCCGTCCAGCTCGACCAG-3′; and human GAPDH: F:5′-TATGACAACAGCCTCAAGAT-3′ and R:5′-AGTCCTTCCACGATACCA-3′.

### Protein isolation and western blotting

A whole protein extraction kit (Solarbio, R0010, Beijing, China) was used to extract cell proteins. Nuclear Protein Extraction Kit (Solarbio, R0050, Beijing, China) was used to extract Nuclear proteins. Primary antibodies against ZNF24, SLC7A5 and β-actin were purchased from Proteintech (11219-1-AP, diluted at 1:1000), Santa Cruz (sc-374232, diluted at 1:500) and ZSGB-BIO (TA-09, diluted at 1:1000), LaminB purchased from ZENBIO (R24828, diluted at 1:1000). Incubated at 4 °C overnight. Secondary antibodies were diluted at a ratio of 1:10000 (ZSGB-BIO, Zhongshan, China), incubated at 37°C for 1h. The final results were detected by a high-efficiency chemiluminescence (ECL) kit (Baisai, P82011, Shanghai, China).

### Immunofluorescence colocalization experiment

Cell monolayers were prepared (Each slide was spread with 5 × 10^4^ cells and placed in an incubator at 37° C for 12-24 hours). 200 µL of 4% paraformaldehyde was dropped onto the cell surface, and the slides were fixed for 30 minutes. The slides were percolated with 0.2% Triton X-100 for 10 min. After blocking with 5% BSA, the primary antibody was incubated at 37°C. SLC7A5 (Mouse monoclonal antibody, sc-374232, diluted at 1:100) and ZNF24 (Rabbit Polyclonal Antibody, 11219-1-AP, Proteintech) antibodies were mixed at a 1:1 ratio and dropped onto slides with 200 µL of antibody dilution. Slides were washed 3 times with PBS. Then, the secondary antibody was added and incubated for 1 hour at 25 °C, and DAPI was added. The photographs were obtained by fluorescence microscopy and analyzed by ImageJ software (Version 1.53e, Wayne Rasband and contributors National Institutes of Health, USA. http://imagej.nih.gov/ij).

### Immunoprecipitation

Cell lysates containing protease inhibitors (R0100, Solarbio, China) were added and incubated for 20-25 min. The protein concentration was determined by the BCA kit (P0012, Beyotime, China) and adjusted to 1 µg/µL. Divided into 1.5ml EP tubes, 500µL/tube. Added IgG antibody, SLC7A5 antibody or ZNF24 antibody (about 1ug), incubated at 4°C overnight. Then, 30 µL of A/G protein beads (20423, *In vivo*Gen) was added to the supernatant, incubated for 4-6 hours, and centrifuged at 2500 rpm for 5 min. After the last wash, the supernatant was removed, and 40 µL of 1X SDS‐PAGE loading buffer was added to the precipitate. After incubation at 98°C for 10 min, a portion or all of the samples were analyzed by SDS‐PAGE and western blotting.

### Pulldown assays

HEK293T cells were cultured, and the cells reached approximately 80%. Plasmid-SLC7A5-myc and plasmid-ZNF24-3×Flag were cotransfected into HEK293T cells. After 36-48 hours of transfection, the cellular protein was collected. A total of 10 or 20 µL of a magnetic bead suspension from a Myc-labeled protein immunoprecipitation kit (P2183S, Beyotime) or Flag-labeled protein immunoprecipitation kit (P2181S, Beyotime) was added to samples containing equal amounts of protein. Then, the samples were placed on their side on a shaking table and incubated at 4 °C overnight. After incubation, the samples were placed on a magnetic frame for separation for 10 seconds to remove the supernatant. Every 20 μ L Original magnetic bead volume, add 100 μL c-Myc peptide eluent or 100ul 3 × Flag peptide eluent. c-Myc peptide eluent (P9805, Beyotime) and 3 × Flag peptide eluent (P9801, Beyotime) storage mother liquor is 5mg/ml TBS (50mM Tris HCl, pH7.4, 150mM NaCl) solution. During operation, use deionized water to dilute the mother liquor to 150 μ g/ml. Evenly mix the magnetic beads with the eluent, place them on a side sway shaker or rotary mixer, and incubate them at room temperature for 30-60 minutes, or at 4 °C for 1-2 hours.). After incubation, the supernatant was separated on the magnetic rack for 10 seconds and transferred to a new centrifuge tube. The supernatant is the eluted tag protein and its complex. The eluted tag protein and its complex are stored at 4 °C for future use, or - 20 °C or - 80 °C for long-term storage.

### Yeast two-hybrid assay

The full-length ORFs of SLC7A5 were amplified by gene-specific primers and then cloned into pGBKT7 vectors. ZNF24 was amplified using gene-specific primers and inserted into pGADT7 vectors (Clontech, http://www.cell-research.com). SLC7A5-pGBKT7 and ZNF24-pGADT7 were transformed into yeast strain AH109 by cotransformation. The positive control pGADT7-large T + pGBKT7-p53 and the negative control pGADT7-large T + pGBKT7-lamin C were resuspended in 2 mL of sterile ddH_2_O, and spots were placed in SD-TL, SD-TLH, SD-TLHA, and SD-TLHA+ x-α-Gal in the plate.

### Cell counting Kit-8 assay

Cells (A549 or H358) were seeded in 96-well plates with 5×10^3^-1×10^4^ cells in each well and cultured at 37°C for 12 hours. 10 µL CCK-8 solution (CA1210, Solarbio) was added to each individual experimental well the next day and incubated at 37°C for 1 hour. A microplate reader (Model 680, Bio-RAD) with a 450 nm absorbance filter was used to read the value.

### Clone formation assay for cell proliferation assessment

A total of 1000 cells per well were seeded on 6-well plates. The medium was changed every 3-4 days, and the culture was terminated after the cells formed visible clones. One milliliter of methanol was added to each well, and the cells were fixed for 15 min. The cells were rinsed with water 3-5 times. Photographs were taken, PS software was used for counting, and the following formula was used: clonal formation rate (%) = clonal coefficient/number of inoculated cells ×100%.

### Cell apoptosis assay

Cells were collected by centrifugation and single-cell suspension was prepared and 1 × 10^5^ cells were added to 200 μL of binding buffer. According to the instructions of the Apoptosis kit (C1062L, Beyotime), Cells were incubated with Annexin V-FITC for 15 min at 25°C, and incubated in the dark with buffer and PI at 4°C for 30 min. Fluorescence detection was performed by flow cytometry.

### TUNEL analysis

Cell sliders were prepared (Each slide was spread with 5 × 10^4^ cells and placed in an incubator at 37° C for 12-24 hours). Apoptosis of A549 and H358 cells was analyzed by a TUNEL assay kit (*In Situ* Cell Death Detection Kit POD; Beyotime, China). 200 µL of 4% paraformaldehyde was dropped onto the cell surface, and the slides were fixed for 30 minutes. The slides were percolated with 0.2% Triton X-100 for 10 min. After washing with PBS for 3 times, 50uL TUNEL reaction solution was added. The samples were incubated for 1 hour at 37°C. After washing with PBS three times, 10uL DAPI was added. The slices were sealed and then examined under a fluorescence microscope.

### Transwell migration and invasion assay

Transwell chambers (CLS3412-24EA, Corning Inc.) was used for migration and invasion tests. A total of 5 × 10^4^ cells were resuspended in serum-free medium containing Matrigel and seeded into the upper chamber of the Transwell chamber, and an appropriate amount of serum-containing medium was added to the lower chamber. After 24 h, cells invading the Transwell’s lower chamber were fixed with 4% paraformaldehyde and stained with Giemsa staining solution. Cells were photographed, and 10 fields were counted for each sample. The steps used to evaluate cell migration were the same as those for cell invasion but without the addition of Matrigel.

### Wound healing assay

A549 or H358 cells were seeded in 6-well plates with 1 × 10^6^ cells/well in medium supplemented with 10% fetal bovine serum. After the density reached 80%, a 50 μL pipette was used to draw a line at the bottom of the 6-well plate where the cells adhered to the wall, and the observation position was marked. The culture was washed twice with PBS. Each group was then photographed at 24, 48 and 72 hours. The healing area of the cells was calculated with ImageJ software.

### Tumor growth and morphological analysis *in vivo*

Fifteen 6-week-old female BALB/C nude mice were randomly divided into 3 groups (Vital River Laboratories, Beijing, China) and fed under specific pathogen-free (SPF) conditions. The cells (1×10^6^ at each spot) were injected subcutaneously into the armpits of mice to form tumors. The change in subcutaneous tumor size was observed every 3 days. Tumor growth was dynamically monitored with calipers and draw the tumor growth curve. Finally, the mice were killed. The tumors were removed and weighed. The tumor tissues were prepared into paraffin-embedded tissues, and the expressions of ZNF24, SLC7A5 and KI-67 were detected by immunohistochemistry. Histological changes of the tumors were evaluated by HE stains.

### Statistical analysis

Data are shown as the mean ± standard deviation. All statistical analyses were performed using SPSS software version 22.0. Comparisons between all groups were performed using one-way analysis of variance (ANOVA) or t test. p < 0.05 indicates statistical significance.

## Results

### The expression levels of ZNF24 and SLC7A5 in KRAS mutant LUAD were both increased and positively correlated

**Figure 1:**
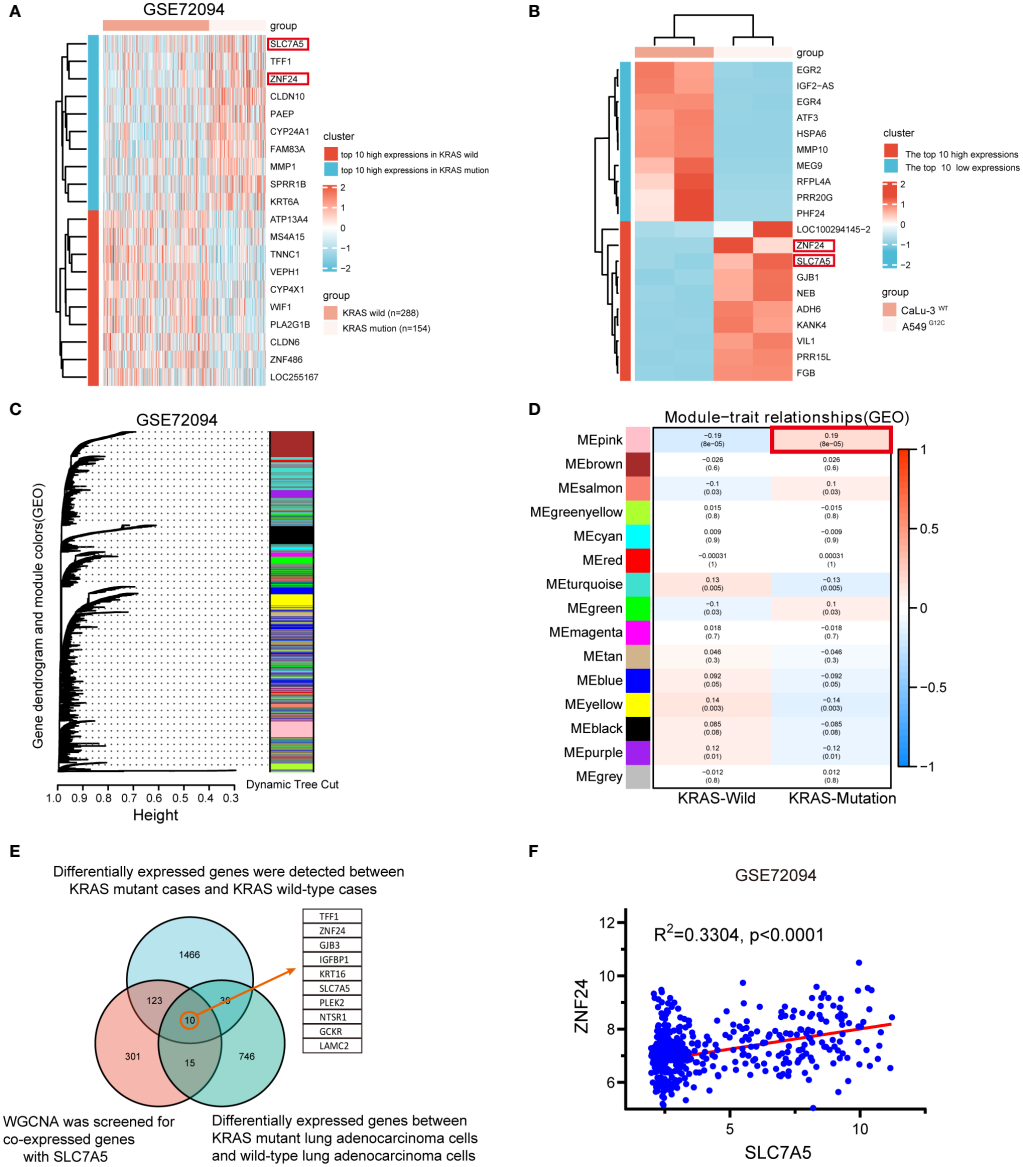
Upregulation and positive correlation of ZNF24 and SLC7A5 in KRAS mutant lung adenocarcinoma. **(A, B)** In the GSE72094 dataset and our RNA-seq, Heatmap of the top 10 high- and low-expression genes; ZNF24 and SLC7A5 are among these high-expression genes. **(C, D)** Weighted gene co-expression network analysis (WGCNA) of the GSE72094 dataset. **(E)** The genes co-expressed with SLC7A5 were screened by WGCNA on the sequencing samples, and the differentially expressed genes were screened in the online database. KRAS-mutant and KRAS wild-type cases were screened, and the genes closely related to SLC7A5 were screened for the intersection of the three sets of data. Take the intersection and draw VENN. **(F)** SLC7A5 positively correlates with ZNF24 expression in KRAS-mutant lung adenocarcinoma in GSE72094.

To further study the pathogenesis of KRAS mutant LUAD, we explored the potential functional genes in LUAD GEO data chips (GSE72094). The expression levels of SLC7A5 and ZNF24 were found to be abnormally elevated in KRAS mutant LUAD tissues compared with KRAS wild-type LUAD tissues (**Figure 1A**, **sFigure 1A**). Next, the KRAS mutant LUAD cell line A549 (KRAS^G12S^) and KRAS wild-type LUAD cell line CaLu-3 were sequenced (**Figure 1B**, **sFigure 1B**). Meanwhile, we carried out weighted gene co-expression network analysis of the GSE72094 dataset. Unsupervised clustering resulted in 15 modules (**Figure 1C**), and correlation analysis was conducted in the 15 modules with KRAS wild-type cases and mutant cases (**Figure 1D**). Based on the above results, a Venn diagram (**Figure 1E**) was generated. The expression levels of SLC7A5 and ZNF24 were both abnormally increased, and the expression levels of the two genes were positively correlated (**Figure 1F**).

**Figure 2:**
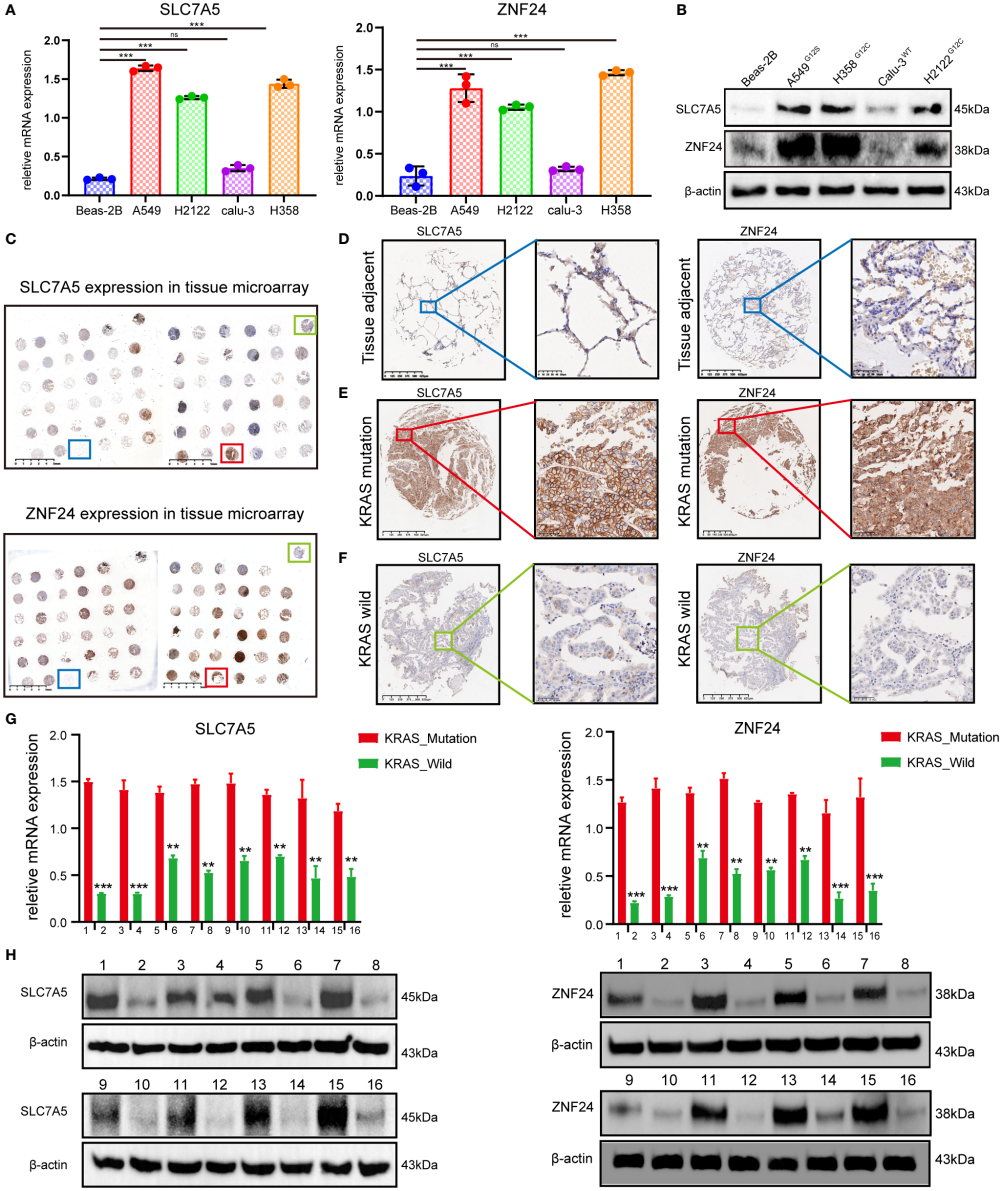
ZNF24 and SLC7A5 are up-regulated in lung adenocarcinoma cell lines and tissues with KRAS mutation. **(A, B)** qRT-PCR and Western blot analysis showed that ZNF24 and SLC7A5 were significantly up-regulated in lung adenocarcinoma cells with KRAS mutation. **(C–F)** Immunohistochemical staining of SLC7A5 and ZNF24 in tissue microarray showed that ZNF24 and SLC7A5 were significantly up-regulated in lung adenocarcinoma tissues with KRAS mutation. **(G, H)** qRT-PCR and Western blot analysis showed that ZNF24 and SLC7A5 were significantly upregulated in lung adenocarcinoma clinical tissue samples with KRAS mutation. The experiments were repeated 3 times. Data are shown as means ± SD. P values were calculated with two-tailed Student’s t-test, *p < 0.05, **p < 0.01, ***p < 0.001. ns, not significant.

To further clarify the expression relationship between SLC7A5 and ZNF24 in KRAS mutant lung adenocarcinoma, we selected KRAS mutant lung adenocarcinoma cell lines (A549^G12S^, H358^G12C^, and H2122^G12C^), the KRAS wild-type LUAD cell line CaLu-3 and the lung normal epithelial cell line Beas-2B to detect the expression levels of ZNF24 and SLC7A5. The results confirmed that the expression levels of both factors were significantly increased in KRAS mutant LUAD cell lines (A549^G12S^, H358^G12C^, and H2122^G12C^) (**Figures 2A, B**). Next, the LUAD clinical specimens were prepared into tissue microarray, and immunohistochemical analysis was performed. As shown in **Figures 2C–F**, ZNF24 and SLC7A5 expression were significantly increased in KRAS mutant lung adenocarcinoma. Then, the expression levels of ZNF24 and SLC7A5 were detected in 16 selected KRAS mutation tumor tissues and KRAS wild-type tumor tissues from the samples by qPCR and Western blot analysis, and we found that the expression levels of ZNF24 and SLC7A5 were both increased in KRAS mutation patients (**Figures 2G–H**).

### Knockdown of ZNF24 reduces the level of nuclear SLC7A5 protein

**Figure 3:**
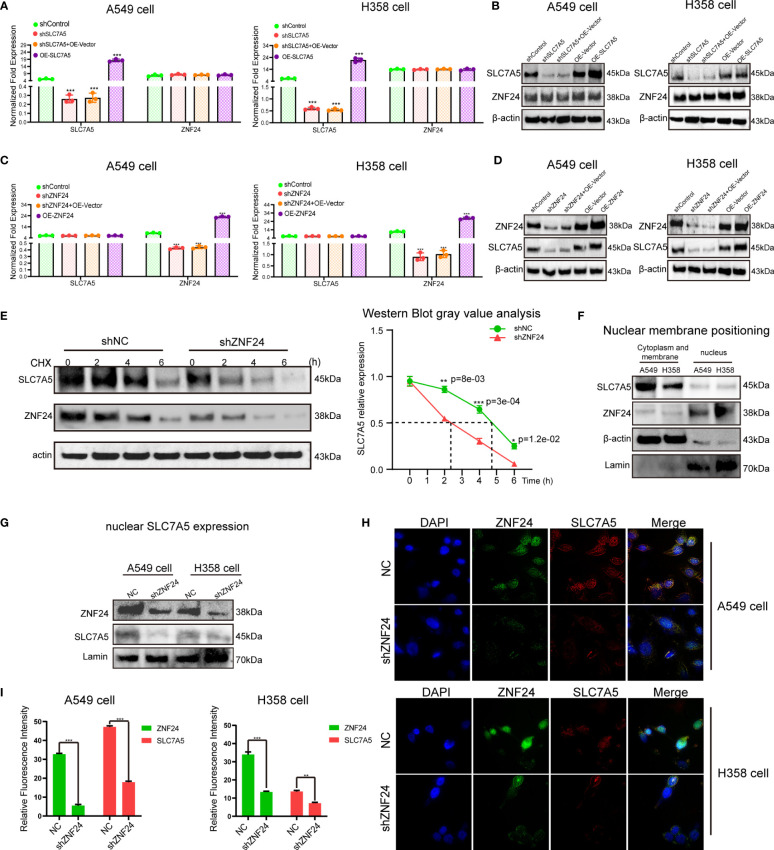
Knockdown of ZNF24 reduces the level of SLC7A5 protein. **(A–D)** The regulation between ZNF24 and SLC7A5 was analyzed by qRT-PCR and Western blot, and ZNF24 positively regulated the protein level of SLC7A5. **(E)** CHX assay showed that ZNF24 knockdown shortened the half-life of SLC7A5 protein synthesis. **(F)** Western blot showed that SLC7A5 was expressed in the cytoplasmic membrane and nuclear of A549 and H358, while ZNF24 was mainly expressed in the nucleus. **(G)** Extract nuclear protein and Western blot showed that knockdown of ZNF24 inhibited the expression of SLC7A5 in the nucleus. **(H, I)**. Immunofluorescence assay showed that SLC7A5 expression was decreased after ZNF24 was knocked out. The experiments were repeated 3 times. Data are shown as means ± SD. P values were calculated with two-tailed Student’s t-test, *p < 0.05, **p < 0.01, ***p < 0.001.

Next, we studied the specific regulatory relationship between ZNF24 and SLC7A5. First, the expression of SLC7A5 was inhibited, and no changes were found in the mRNA and protein levels of ZNF24 (**Figures 3A, B**). Then, we observed that ZNF24 knockdown did not affect SLC7A5 transcription (**Figure 3C**). However, ZNF24 knockdown significantly downregulated SLC7A5 protein expression levels (**Figure 3D**). These data suggested that ZNF24 has a positive regulatory effect on SLC7A5 protein levels but has no significant regulatory effect on SLC7A5 transcription. In addition, the half-life of SLC7A5 was shortened, and the degradation rate was accelerated after actinomycin (CHX) treatment, which affected the overall protein level (**Figure 3E**).

We detected SLC7A5 localized in the nucleus and membrane and ZNF24 localized in the nucleus (**Figure 3F**). Since ZNF24 knockdown can inhibit SLC7A5 translation and reduce SLC7A5 protein expression levels in whole cells, we speculated that ZNF24 has the ability to reduce nuclear SLC7A5 activity. As expected, knockdown of ZNF24 significantly inhibited SLC7A5 expression (**Figure 3G**) in the nucleus, as shown by Western blotting. Then, immunofluorescence analysis showed that the protein expression level of SLC7A5 in the nucleus was remarkably reduced after ZNF24 knockdown (**Figures 3H, I**). These experiments demonstrated that ZNF24 knockdown could reduce the level of nuclear SLC7A5.

### An endogenous protein interaction between ZNF24 and SLC7A5 occurred

**Figure 4:**
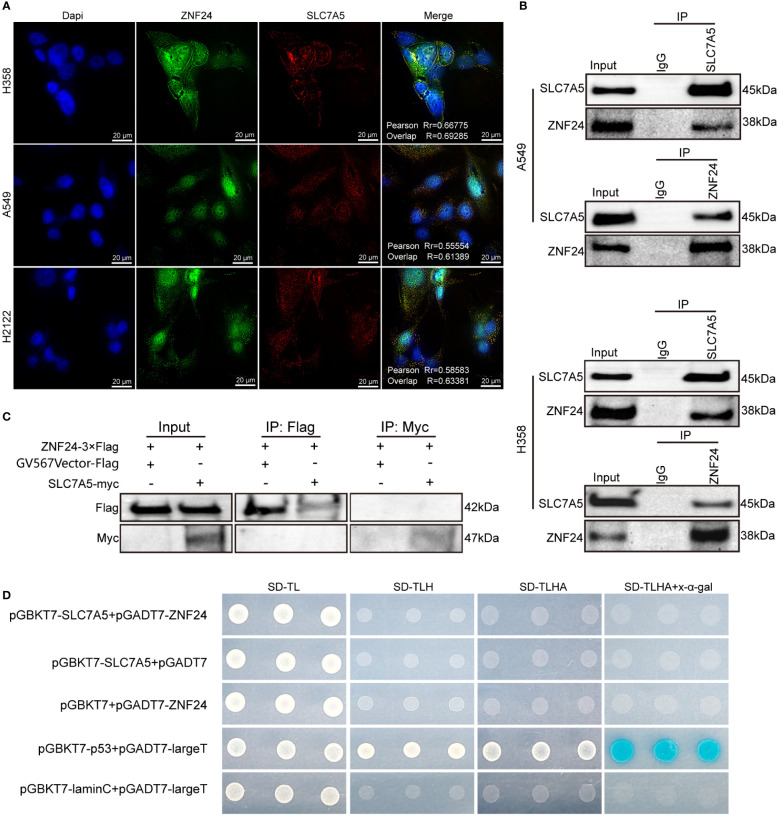
An endogenous protein interaction between ZNF24 and SLC7A5 occurred. **(A)** Immunofluorescence assay showed colocalization of SLC7A5 and ZNF24 in KRAS mutant lung adenocarcinoma cells. **(B)** Cell lysates from A549 and H358 cells were separately analyzed by IP and Western blotting using the indicated antibodies, ZNF24 interacted with SLC7A5. A representative image is shown, n = 3 independent experiments. **(C)** Pull-down assay showed no direct protein interaction between SLC7A5 and ZNF24. **(D)** Yeast two-hybrid assay showed no direct protein interaction between SLC7A5 and ZNF24.

Since ZNF24 can regulate the SLC7A5 protein expression level, we hypothesized that ZNF24 interacts with SLC7A5. Immunofluorescence experiments further confirmed the colocalization of ZNF24 and SLC7A5 (**Figure 4A**). Next, we performed immunoprecipitation assays in H358 and A549 cell lines, and the results showed that ZNF24 interacted with SLC7A5 endogenous protein (**Figure 4B**). To further clarify whether there was a direct protein interaction between ZNF24 and SLC7A5, we carried out yeast two-hybrid and pull-down experiments, and the results showed that no direct protein interaction occurred between ZNF24 and SLC7A5 (**Figures 4C, D**). This result suggested that there were other bridge proteins between ZNF24 and SLC7A5. Our experiment lays a foundation for further research on the regulatory relationship between these two factors.

### Oncogenic RAS signaling regulates ZNF24 and SLC7A5 expression through MEK-ERK and PI3K-AKT pathways in KRAS mutant lung adenocarcinoma

**Figure 5:**
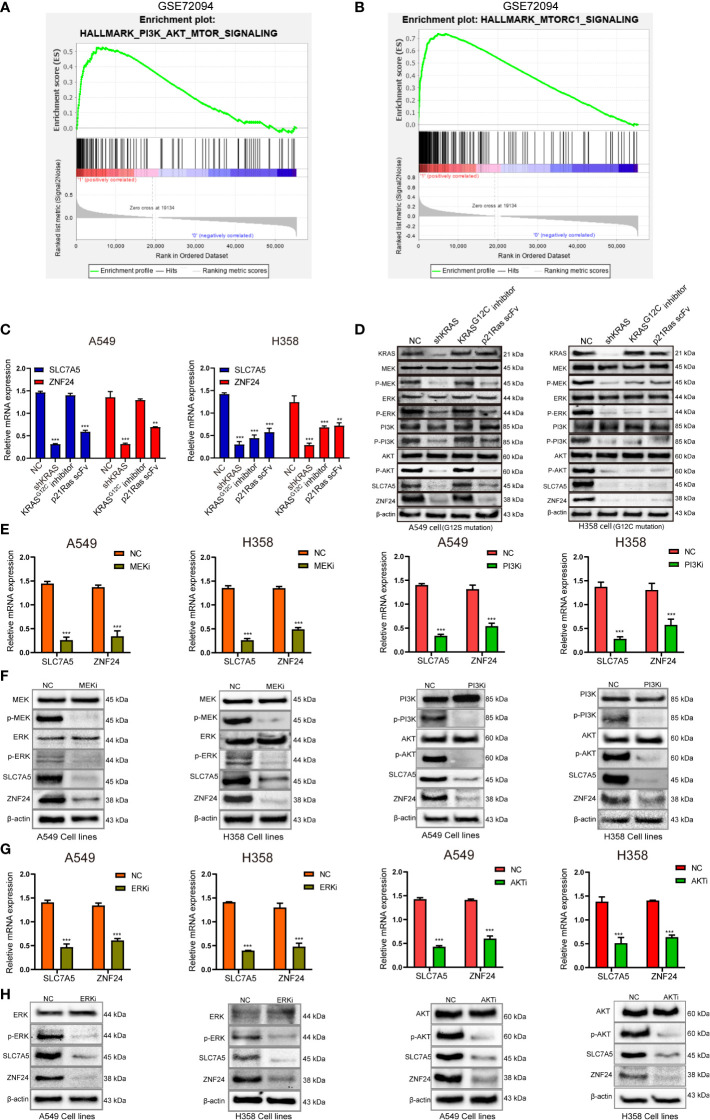
Oncogenic RAS signaling regulates ZNF24 and SLC7A5 expression through MEK-ERK and PI3K-AKT pathways in KRAS mutant lung adenocarcinoma. **(A, B)** Gene set enrichment analysis (GSEA) diagram showing that SLC7A5 was enriched in the PI3K-AKT-mTOR signaling pathway. **(C, D)** qRT-PCR and Western blot results showed that the expression of SLC7A5 and ZNF24 was down-regulated after KRAS blockade. **(E, F)** The results of qRT-PCR and Western blot showed that the expression of SLC7A5 and ZNF24 was down-regulated after inhibiting MEK and PI3K. **(G, H)** The results of qRT-PCR and Western blot showed that the expression of SLC7A5 and ZNF24 was down-regulated after inhibiting ERK and AKT. The experiments were repeated 3 times. Abbreviations: shRNA, short hairpin RNA; NC, negative control. Data are shown as means ± SD. P values were calculated with two-tailed paired Student’s t-test, *p < 0.05, **p < 0.01, ***p < 0.001.

Gene set enrichment analysis showed that SLC7A5 was enriched in the PI3K-AKT-mTOR pathway downstream of RAS (**Figures 5A, B**). We attempted to investigate whether ZNF24 and SLC7A5 also participate in the MEK-ERK signaling pathway to determine the downstream pathway through which RAS mutations regulate ZNF24 and SLC7A5. To investigate whether ZNF24 and SLC7A5 are regulated by MEK-ERK and PI3K-AKT downstream of RAS, we first used different methods (KRAS^G12C^inhibitor HY-18707, small interfering RNA, RGD-p21Ras-scFv) to interfere with KRAS expression and discovered that the protein and mRNA expression levels of ZNF24 and SLC7A5 were markedly downregulated (**Figures 5C, D**). Subsequently, MEK- and PI3K-specific inhibitors were used to block downstream RAS pathways, and the mRNA and protein expression levels of ZNF24 and SLC7A5 were sharply downregulated (**Figures 5E, F**). Downstream of MEK and PI3K, the inhibition of ERK1/2 and AKT effectively reduced the expression of ZNF24 and SLC7A5 (**Figures 5G, H**).

The above results indicated that the oncogenic RAS signaling pathways *via* MEK-ERK and PI3K-Akt were sufficient to drive the expression of ZNF24 and SLC7A5. Since RAS signaling is closely related to malignant tumor proliferation, we hypothesized that ZNF24 may promote the progression of KRAS mutant LUAD by upregulating SLC7A5 protein expression.

### ZNF24 promotes KRAS mutant LUAD cell growth through SLC7A5

First, we investigated the effect of SLC7A5 on the biological behavior of KRAS mutant LUAD cell lines. The results showed that after SLC7A5 knockdown, proliferation was decreased (**sFigures 2A, B**), while the invasion and migration abilities of A549 and H358 cells were markedly decreased (**sFigure 2C**).

**Figure 6:**
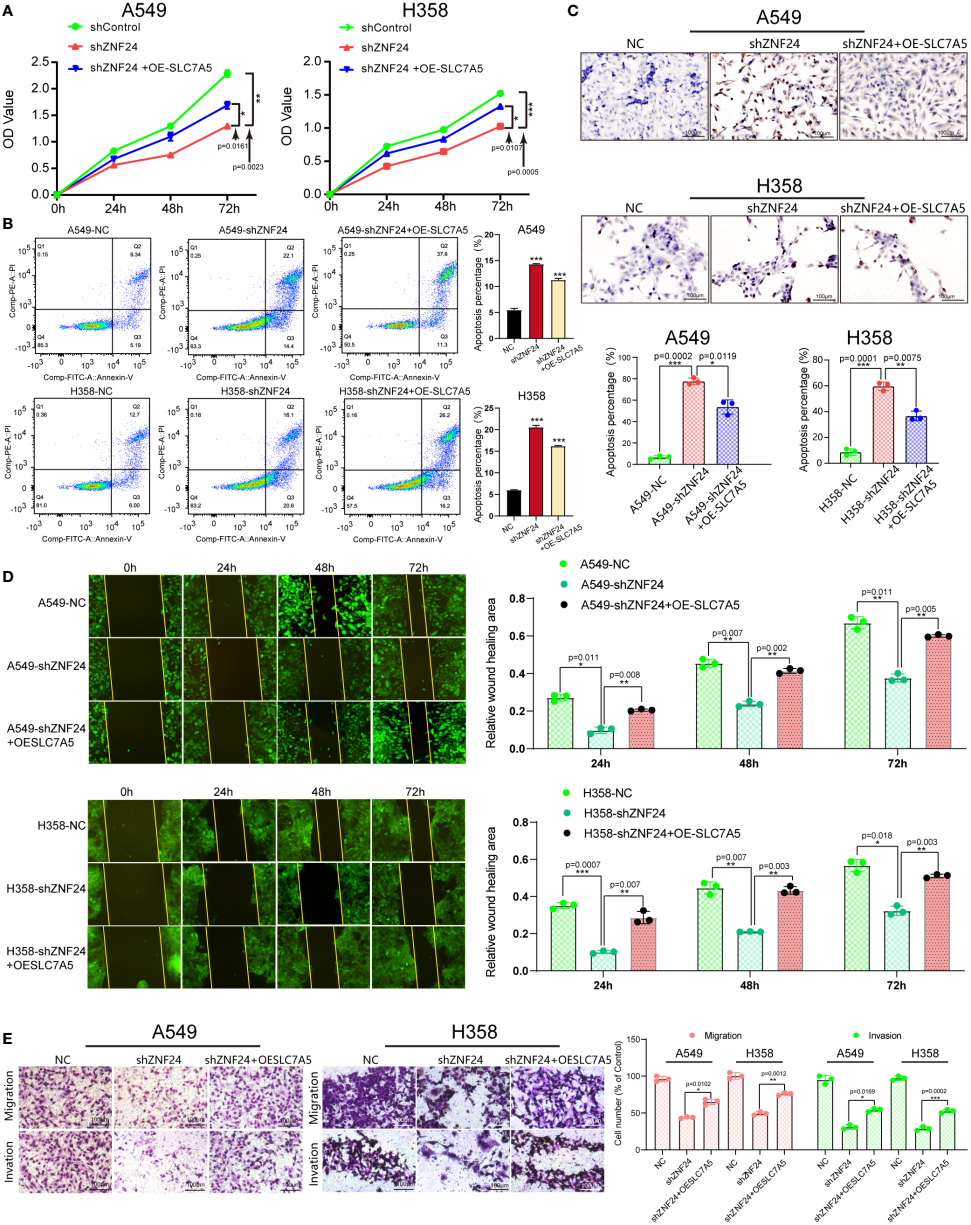
ZNF24 promotes KRAS mutant LUAD cell growth through SLC7A5. **(A)** The CCK8 assay was used to detect cell proliferation. **(B, C)** Cell apoptosis was detected by flow cytometry and the TUNEL assay. **(D)** The migration ability of cells was measured by the wound healing assay. **(E)** Transwell assays were used to measure the invasion ability of cells. The experiments were repeated 3 times. shRNA, short hairpin RNA; NC, negative control; OE, overexpression. Data are shown as means ± SD. P values were calculated with two-tailed paired Student’s t-test, *p < 0.05, **p < 0.01, ***p < 0.001.

Next, we investigated whether ZNF24 could affect the biological behavior of KRAS mutant LUAD cells through SLC7A5. CCK8 assay results showed that the cell viability decreased significantly after ZNF24 knockdown, while SLC7A5 was overexpressed after ZNF24 knockdown, and the cell viability recovered significantly. This result was similar to that of the untreated cancer cells (H358, A549) (**Figure 6A**), suggesting that ZNF24 promoted the proliferation of KRAS mutant LUAD cells through SLC7A5. The results of flow cytometry and TUNEL assays further indicated that the number of apoptotic cells overexpressing SLC7A5 decreased after ZNF24 knockdown (**Figures 6B, C**). Wound healing and transwell assays also demonstrated that ZNF24 promoted the migration and invasion of KRAS mutant LUAD cells *via* SLC7A5 (**Figures 6D, E**).

### ZNF24 promotes the growth of KRAS mutant LUAD grafts *via* SLC7A5

As ZNF24 has been proven to promote the growth of SLC7A5-mediated KRAS mutant LUAD cells *in vitro*, we established a nude mouse transplanted tumor model for *in vivo* experimental verification. The results showed that SLC7A5 fluorescence intensity was significantly decreased (**sFigure 2D**), and the growth rate of the tumor was slower than that of the control group (**sFigures 2E–G**). Immunohistochemical results further showed that SLC7A5 significantly affected the growth of LUAD (**sFigure 2H**).

**Figure 7:**
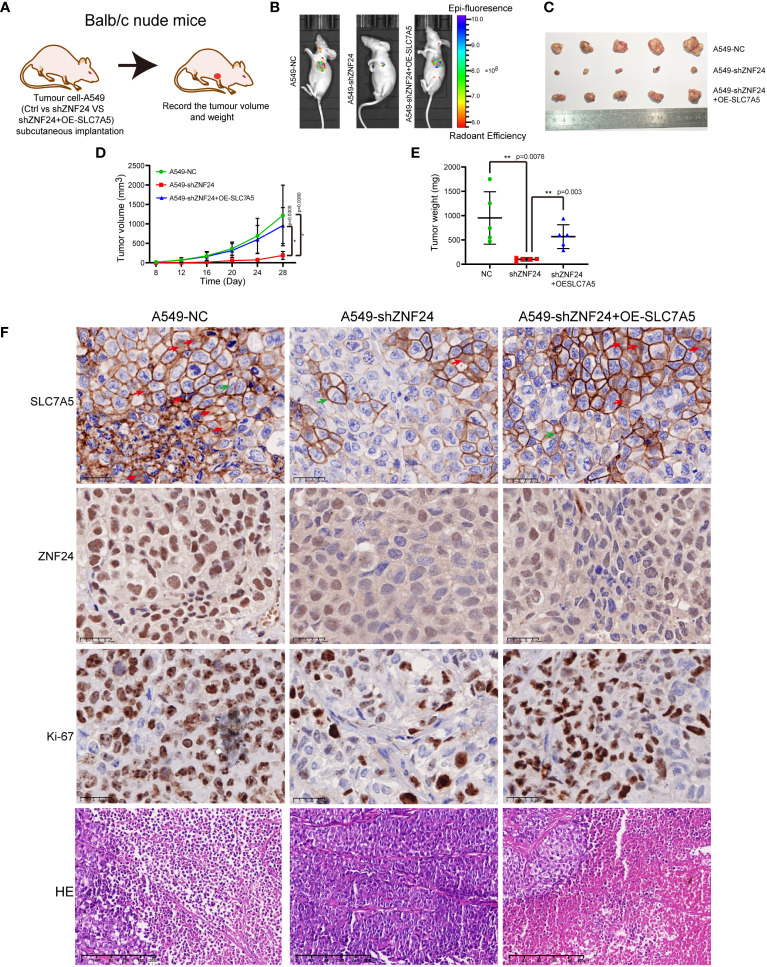
ZNF24 promotes the growth of KRAS mutant lung adenocarcinoma grafts *via* SLC7A5. **(A)** Transplanted tumor model in nude mice by subcutaneous injection of tumor cells. **(B)** *In vivo* imaging of mice showed that the tumor growth rate was significantly delayed after knockdown of ZNF24, and the inhibition of ZNF24 could be partially reversed after recovery of SLC7A5. **(C–E)** Measurement of the primary tumor volume and weight. ZNF24 promoted tumor growth through SLC7A5. **(F)**, HE staining showed focal necrosis of cells in shZNF24 group but massive necrosis of cells in NC and shZNF24+OE-SLC7A5 groups. Immunohistochemical staining showed that the SLC7A5 was expressed in membrane (green arrow) and nucleus (red arrow) of tumor, and the expression was significantly less in shZNF24 group than in NC and shZNF24+OE-SLC7A5 groups. There were less Ki-67 positive tumor cells in shZNF24 group than in NC and shZNF24+OE-SLC7A5 groups. NC, negative control; OE, overexpression. Data are shown as means ± SD. P values were calculated with two-tailed paired Student’s t-test, *p < 0.05, **p < 0.01, ***p < 0.001.

Next, we demonstrated that ZNF24 promoted the growth of KRAS mutant LUAD transplantation tumors *via* SLC7A5 (**Figure 7A**). *In vivo* imaging of mice confirmed that the fluorescence intensity of the NC group was higher than that of the ZNF24 knockdown group, and the fluorescence intensity of the SLC7A5 overexpression group after ZNF24 knockdown was significantly higher than that of the ZNF24 knockdown group (**Figure 7B**). By plotting the tumor growth curve and comparing tumor volumes, we observed that knocking down ZNF24 significantly inhibited the growth of A549 cell-transplanted tumors, while SLC7A5 overexpression after ZNF24 knockdown partially reversed tumor inhibition (**Figures 7C–E**). In shZNF24 group, HE staining showed focal necrosis of xenograft tumor. In NC and shZNF24+OE-SLC7A5 groups, the results showed massive necrosis of the tumor. Immunohistochemistry demonstrated that the expression of SLC7A5 was significantly downregulated in shZNF24 group. In addition, knockdown of ZNF24 reduced ki-67 positive cells in transplanted tumors, while SLC7A5 overexpression after ZNF24 knockdown partially reversed tumor suppression, and ki-67 positive cells were increased (**Figure 7F**).

## Discussion

RAS is one of the fundamental drivers of LUAD (15, 16). RAS plays a core role in mitotic signal transduction, and, the downstream effector factors are involved in the activation of mitotic pathways, which are related to tumorigenesis (17–19). Current therapies include Ras-direct inhibitors and inhibitors of upstream or downstream signaling pathways (20, 21). However, due to the escape inhibition, extratarget toxicity and single action site of these inhibitors, research and application have been greatly limited. For example, Sotorasib, a Kras inhibitor targeting G12C, has been approved by the FDA for marketing, but it is only effective for G12C mutation site, rather than other mutation sites, and the objective remission rate is only 32% (22). There are many factors that affect the objective remission rate of drugs, and the mutation of downstream genes can also affect the effect of upstream gene targeted therapy. For example, BRAF mutations in the downstream of EGRF can affect the therapeutic effect of TKI inhibitor targeting EGFR (23). Ras signal pathway includes many genes, and mutations in downstream genes may also affect the therapeutic effect of Ras inhibitors. Therefore, our study aimed to explore the regulatory factors and mechanisms of KRAS mutant LUAD from the perspective of blocking the Ras signaling pathway and antagonizing relevant influencing molecules, explore new therapeutic targets and provide new guidance for targeted tumor therapies.

In our study, KRAS mutant LUAD cases were selected for transcriptome sequencing of KRAS mutant LUAD cell lines, and differentially expressed genes in KRAS mutant LUAD were identified by integrated bioinformatics analysis (**Figure 1**, **sFigure 1**) and verified by qRT‐PCR, WB, and immunohistochemistry. ZNF24 and SLC7A5 were identified as candidate target genes. The expression of the two genes was significantly correlated, and the high expression of both genes was closely linked to poor prognosis (**Figure 2**). These two abnormally expressed genes were first discovered in LUAD with KRAS mutation. It is of great significance to identify these two factors as candidate target genes and further explore their regulatory relationship for further investigation of KRAS mutant LUAD. To our surprise, a recent study showed that KRAS mutation in colorectal cancer drives upregulation of SLC7A5, which provides strong support for our target screening results.

SLC7A5, as one of the main drivers of tumorigenesis, promotes protein synthesis in cancer cells by regulating the mTORC1 signaling pathway and the general amino acid control (GAAC) pathway (24–26). In this study, lentivirus carrying shRNA-SLC7A5 was transfected into H358 and A549 cell lines, demonstrating that SLC7A5 knockdown could suppress the migration, proliferation, and invasion of KRAS mutant LUAD cells and induce apoptosis. *In vivo* experiments further showed that downregulation of SLC7A5 inhibited tumor growth (**sFigure 2**) All of these results confirmed that SLC7A5, as an oncogenic gene, can mediate the development of KRAS mutant tumors. However, the mechanism of SLC7A5-specific enrichment in KRAS mutant LUAD is still unknown, and its regulatory relationship with ZNF24 has not been reported. In this study, SLC7A5 and ZNF24 were knocked down, and ZNF24 was identified as the positive regulator of SLC7A5, which inhibited SLC7A5 expression at the protein level. SLC7A5 was expressed in the nucleus during the experiment, and ZNF24 knockdown reduced the expression of SLC7A5 in the nucleus (**Figure 3**). These results also suggested that SLC7A5 and ZNF24 underwent protein interactions and that the main regulatory interaction of the two proteins occurred in the nucleus. These experimental results motivated us to perform further investigations. Subsequent experiments confirmed our hypothesis that SLC7A5 and ZNF24 had indirect protein interactions (**Figure 4**). ZNF24 may form a cofactor with a ubiquitinated protein to further regulate the expression of SLC7A5. This ubiquitinated protein is of key significance for the connection between ZNF24 and SLC7A5, but further studies need to be performed in the future. The ZNF24-SLC7A5 signaling axis established in this study further explains the oncogenic mechanism of SLC7A5. In view of the urgent need for in-depth exploration of downstream targets of Ras to address the difficult problem of Ras targeting (27, 28) and drug resistance in the treatment of Ras mutant LUAD (29–31), the elucidation of ZNF24 greatly aids research on the above problems.

ZNF24 was first discovered in 1998 (32), and relevant studies have confirmed that knocking out ZNF24 can lead to premature death at different time points during development, suggesting that ZNF24 plays a key role in regulating organ development (33). As a transcription factor, ZNF24 plays an inhibitory role in breast and gastric cancers (34, 35), but it plays a role as an oncogene in prostate and liver cancers (13, 14, 36). However, the role of ZNF24 in KRAS mutant LUAD and its role in the RAS signaling pathway have not yet been reported. By blocking RAS and its downstream MEK/ERK and PI3K/AKT signaling pathways, our study proved for the first time that SLC7A5 and ZNF24 were positively regulated by the RAS signaling pathway, and it showed that SLC7A5 and ZNF24 were located downstream of MEK/ERK and PI3K/AKT and were new members of the RAS signaling pathway (**Figure 5**). Our study confirmed the existence of the RAS-ZNF24-SLC7A5 signaling axis in detail and revealed the regulatory relationship between ZNF24 and SLC7A5. Meanwhile, *in vivo* and *in vitro* experiments also demonstrated that ZNF24 could induce the growth of KRAS mutant LUAD through SLC7A5 (**Figure 6** and **Figure 7**). These studies are the first to elucidate the role of ZNF24 in KRAS mutant LUAD. A new target provided by our work for the treatment of RAS signaling pathway and a supplement to the current inhibition targets.

In addition, since the upregulation of SLC7A5 is closely related to abnormal amino acid metabolism (9) and immune evasion (37, 38), in this study, we identified the regulatory mechanism of ZNF24 on SLC7A5, which is crucial for the blockade of tumor metabolism and immunotherapy of KRAS mutant LUAD. We hypothesized that screening small-molecule inhibitors of ZNF24 and combining them with the p21Ras-scFv (an antibody to Ras, previously developed by our research group) (39–42) would have a reverse effect on the progression of Ras mutant LUAD, and these assumptions need to be verified by our group in the future.

## Conclusions

ZNF24 promoted the growth of KRAS mutant LUAD by upregulating SLC7A5 protein expression, which suggests that ZNF24 is a new biomarker of KRAS mutant tumors and could be a new potential therapeutic target for Ras-driven tumors.

## Data availability statement

The datasets presented in this study can be found in online repositories. The names of the repository/repositories and accession number(s) can be found in the article/**Supplementary Material**.

## Ethics statement

This study was supported by the Ethics Committee of the 920th Hospital of the Joint Logistic Support Force of the People’s Liberation Army, with approval number 2022-013-01. Specimens were handled in accordance with legal and ethical regulations. The patients/participants provided their written informed consent to participate in this study. This study was supported by the Ethics Committee of the 920th Hospital of the Joint Logistic Support Force of the People’s Liberation Army, with approval number 2022-013-01. Specimens were handled in accordance with legal and ethical regulations. Written informed consent was obtained from the individual(s) for the publication of any potentially identifiable images or data included in this article.

## Author contributions

The concept and design of this study were provided by DJ, PW and JY. PW, DJ and LL conducted the research and data analysis. The collected experimental data were completed by PL, QF and XP and analyzed by SS and LY. DJ and LL made the manuscript. WP, LL and JY conducted the data review and manuscript review. All authors contributed to the article and approved the submitted version.

## Funding

Major Project of Yunnan Province (2018ZF009).

## Acknowledgments

We thank all authors for their contributions.

## Conflict of interest

The authors declare that the research was conducted in the absence of any commercial or financial relationships that could be construed as a potential conflict of interest.

## Publisher’s note

All claims expressed in this article are solely those of the authors and do not necessarily represent those of their affiliated organizations, or those of the publisher, the editors and the reviewers. Any product that may be evaluated in this article, or claim that may be made by its manufacturer, is not guaranteed or endorsed by the publisher.

## Supplementary material

Supplementary Figure 1 After screening for differentially expressed genes, ZNF24 and SLC7A5 were identified as highly expressed genes. **(A)** Volcano map of differentially expressed genes in the KRAS wild-type groups vs. KRAS mutant groups. **(B)** Volcano map of differentially expressed genes in shKRAS vs. NC. The threshold of fold change (FC) was set as |log_2_FC|> 1, *P* < 0.05.

Supplementary Figure 2 Knockdown of SLC7A5 inhibits the growth of KRAS-mutated LUAD cells *in vivo* and *in vitro*. **(A, B)** CCK8 and clone formation assays were performed to detect cell proliferation. **(C)** Transwell assays were used to measure the invasion ability of cells. **(D)** Fluorescence imaging of nude mice. **(E–G)** Measurement of primary tumor volume and weight. **(H)** Immunohistochemical staining for SLC7A5. shRNA, short hairpin RNA; NC, negative control. Data are shown as means ± SD. P values were calculated with two-tailed Student’s t-test, **p* < 0.05, ***p* < 0.01, ****p* < 0.001.

## Abbreviations

LUAD, lung adenocarcinoma; SLC7A5, solute carrier family 7 member 5; qRT‐PCR, quantitative reverse transcription-polymerase chain reaction; WB, Western blot; SDS‐PAGE, sodium dodecyl sulfate‐polyacrylamide gel electrophoresis; CCK-8, Cell Counting Kit-8; IHC, immunohistochemistry; WGCNA, weighted gene coexpression network analysis; ECL, enhanced chemiluminescence; IF, immunofluorescence; GEO, gene expression omnibus.

## References

[1] Kentaro I. Clinicopathological characteristics and mutations driving development of early lung adenocarcinoma: Tumor initiation and progression. Int J Mol Sci (2018) 19(4):1259. doi: 10.3390/ijms19041259 29690599 PMC5979290

[2] Siegel RL Miller KD Fuchs HE Jemal A. Cancer statistics, 2022. CA: A Cancer J Clin (2022) 72(1):7–33. doi: 10.3322/caac.21708 35020204

[3] Siegel RL Miller KD Jemal A. Cancer statistics, 2020. CA: A Cancer J Clin (2020) 70(1):7–30. doi: 10.3322/caac.21590 31912902

[4] Aran V Zalis M Montella T de Sousa CAM Ferrari BL Gil Ferreira C. Evaluation of KRAS concomitant mutations in advanced lung adenocarcinoma patients. Medicina (2021) 57(10):1039. doi: 10.3390/medicina57101039 34684076 PMC8539053

[5] Ricciuti B Leonardi GC Metro G Grignani F Paglialunga L Bellezza G. Targeting the KRAS variant for treatment of non-small cell lung cancer: potential therapeutic applications. Expert Rev Respir Med (2016) 10(1):53–68. doi: 10.1586/17476348.2016.1115349 26714748

[6] Christensen JG Olson P Briere T Wiel C Bergo MO. Targeting Krasg12c-mutant cancer with a mutation-specific inhibitor. J Internal Med (2020) 288(2):183–91. doi: 10.1111/joim.13057 32176377

[7] Tsai FD Lopes MS Zhou M Court H Ponce O Fiordalisi JJ. K-Ras4A splice variant is widely expressed in cancer and uses a hybrid membrane-targeting motif. Proc Natl Acad Sci (2015) 112(3):779–84. doi: 10.1073/pnas.1412811112 PMC4311840 25561545

[8] Ostrem JM Peters U Sos ML Wells JA Shokat KM. K-Ras (G12C) inhibitors allosterically control GTP affinity and effector interactions. Nature (2013) 503(7477):548–51. doi: 10.1038/nature12796 PMC4274051 24256730

[9] Scalise M Galluccio M Console L Pochini L Indiveri C. The human SLC7A5 (LAT1): the intriguing histidine/large neutral amino acid transporter and its relevance to human health. Front Chem (2018) 6:243. doi: 10.3389/fchem.2018.00243 29988369 PMC6023973

[10] Zhang J Xu Y Li D Fu L Zhang X Bao Y. Review of the correlation of LAT1 with diseases: mechanism and treatment. Front Chem (2020) 8:564809. doi: 10.3389/fchem.2020.564809 33195053 PMC7606929

[11] Kaira K Toyoda M Shino M Sakakura K Takahashi K Tominaga H. Clinicopathological significance of l-type amino acid transporter 1 (LAT1) expression in patients with adenoid cystic carcinoma. Pathol Oncol Res (2013) 19(4):649–56. doi: 10.1007/s12253-013-9624-2 23516127

[12] Najumudeen AK Ceteci F Fey SK Hamm G Steven RT Hall H. The amino acid transporter SLC7A5 is required for efficient growth of KRAS-mutant colorectal cancer. Nat Genet (2021) 53(1):16–26. doi: 10.1038/s41588-020-00753-3 33414552

[13] Huang X Liu N Xiong X. ZNF24 is upregulated in prostate cancer and facilitates the epithelial−to−mesenchymal transition through the regulation of Twist1. Oncol Lett (2020) 19(5):3593–601. doi: 10.3892/ol.2020.11456 PMC7115158 32269634

[14] Liu G Jiang S Wang C Jiang W Liu Z Liu C. Zinc finger transcription factor 191, directly binding to β-catenin promoter, promotes cell proliferation of hepatocellular carcinoma. Hepatology (2012) 55(6):1830–9. doi: 10.1002/hep.25564 22213192

[15] Chen S Li F Xu D Hou K Fang W Li Y. The function of RAS mutation in cancer and advances in its drug research. Curr Pharm Design (2019) 25(10):1105–14. doi: 10.2174/1381612825666190506122228 31057104

[16] Ryan MB Corcoran RB. Therapeutic strategies to target RAS-mutant cancers. Nat Rev Clin Oncol (2018) 15(11):709–20. doi: 10.1038/s41571-018-0105-0 30275515

[17] Castellano E Downward J. RAS interaction with PI3K: more than just another effector pathway. Genes Cancer (2011) 2(3):261–74. doi: 10.1177/1947601911408079 PMC3128635 21779497

[18] Ferrer I Zugazagoitia J Herbertz S John W Paz-Ares L Schmid-Bindert G. KRAS-mutant non-small cell lung cancer: From biology to therapy. Lung cancer. (2018) 124:53–64. doi: 10.1016/j.lungcan.2018.07.013 30268480

[19] Molina JR Adjei AA. The ras/raf/mapk pathway. J Thorac Oncol (2006) 1(1):7–9. doi: 10.1016/S1556-0864(15)31506-9 17409820

[20] Friday BB Adjei AA. Advances in targeting the Ras/Raf/MEK/Erk mitogen-activated protein kinase cascade with MEK inhibitors for cancer therapy. Clin Cancer Res (2008) 14(2):342–6. doi: 10.1158/1078-0432.CCR-07-4790 18223206

[21] Soulières D Greer W Magliocco AM Huntsman D Young S Tsao M-S. KRAS mutation testing in the treatment of metastatic colorectal cancer with anti-EGFR therapies. Curr Oncol (2010) 17(s1):31–40. doi: 10.3747/co.v17is1.614 PMC2901795 20680106

[22] Hong DS Fakih MG Strickler JH Desai J Durm GA Shapiro GI. KRAS(G12C) inhibition with sotorasib in advanced solid tumors. New Engl J Med (2020) 383(13):1207–17. doi: 10.1056/NEJMoa1917239 PMC7571518 32955176

[23] Wang W Gu X Si J Pu X Wang L Chen H. Treatment outcomes and prognosis of patients with primary and acquired BRAF-mutated non-small cell lung cancer: A multicenter retrospective study. Genes Chromosomes Cancer (2022) 61(9):530–41. doi: 10.1002/gcc.23043 35396765

[24] Cheng L Lu W Kulkarni B Pejovic T Yan X Chiang J-H. Analysis of chemotherapy response programs in ovarian cancers by the next-generation sequencing technologies. Gynecologic Oncol (2010) 117(2):159–69. doi: 10.1016/j.ygyno.2010.01.041 PMC2849907 20181382

[25] Kanai Y. Amino acid transporter LAT1 (SLC7A5) as a molecular target for cancer diagnosis and therapeutics. Pharmacol Ther (2022) 230:107964. doi: 10.1016/j.pharmthera.2021.107964 34390745

[26] Wang Q Bailey CG Ng C Tiffen J Thoeng A Minhas V. Androgen receptor and nutrient signaling pathways coordinate the demand for increased amino acid transport during prostate cancer progression. Cancer Res (2011) 71(24):7525–36. doi: 10.1158/0008-5472.CAN-11-1821 22007000

[27] Baines AT Xu D Der CJ. Inhibition of ras for cancer treatment: the search continues. Future Medicinal Chem (2011) 3(14):1787–808. doi: 10.4155/fmc.11.121 PMC3347641 22004085

[28] Marín-Ramos NI Ortega-Gutiérrez S López-Rodríguez ML eds. Blocking Ras inhibition as an antitumor strategy. Semin Cancer Biol (2019) 54:91–100. doi: 10.1016/j.semcancer.2018.01.017 29409706

[29] van Jaarsveld M van Kuijk PF Boersma AW Helleman J van IJcken WF Mathijssen RH. miR-634 restores drug sensitivity in resistant ovarian cancer cells by targeting the ras-MAPK pathway. Mol Cancer (2015) 14(1):1–13. doi: 10.1186/s12943-015-0464-4 26576679 PMC4650519

[30] Zhu S Xu Y Wang L Liao S Wang Y Shi M. Ceramide kinase mediates intrinsic resistance and inferior response to chemotherapy in triple-negative breast cancer by upregulating Ras/ERK and PI3K/Akt pathways. Cancer Cell Int (2021) 21(1):1–11. doi: 10.1186/s12935-020-01735-5 33430896 PMC7802356

[31] Zhu Y Hu Q Li H. Isoprenylcysteine carboxylmethyltransferase is associated with nasopharyngeal carcinoma chemoresistance and ras activation. Biochem Biophys Res Commun (2019) 516(3):784–9. doi: 10.1016/j.bbrc.2019.06.074 31253403

[32] Shi S Liu M Yu L Chen S Zheng Q Wu G. Assignment of a novel zinc finger gene ZNF191 to human chromosome 18Q12. 1 by human/rodent somatic cell hybrid panel and fluorescent *in situ* hybridization. Shi yan Sheng wu xue bao. (1998) 31(1):21–7. 12014109

[33] Li J Chen X Yang H Wang S Guo B Yu L. The zinc finger transcription factor 191 is required for early embryonic development and cell proliferation. Exp Cell Res (2006) 312(20):3990–8. doi: 10.1016/j.yexcr.2006.08.020 17064688

[34] Huang M Huang X Jiang B Zhang P Guo L Cui X. linc00174-EZH2-ZNF24/Runx1-VEGFA regulatory mechanism modulates post-burn wound healing. Mol Therapy-Nucleic Acids (2020) 21:824–36. doi: 10.1016/j.omtn.2020.07.010 PMC7452087 32805486

[35] Liu X Ge X Zhang Z Zhang X Chang J Wu Z. MicroRNA-940 promotes tumor cell invasion and metastasis by downregulating ZNF24 in gastric cancer. Oncotarget (2015) 6(28):25418. doi: 10.18632/oncotarget.4456 26317898 PMC4694841

[36] Liu Y Cheng H Cheng C Zheng F Zhao Z Chen Q. ZNF191 alters DNA methylation and activates the PI3K-AKT pathway in hepatoma cells *via* transcriptional regulation of DNMT1. Cancer Med (2022) 11(5):1269–80. doi: 10.1002/cam4.4535 PMC8894703 35092191

[37] Ansari RE Craze ML Althobiti M Alfarsi L Ellis IO Rakha EA. Enhanced glutamine uptake influences composition of immune cell infiltrates in breast cancer. Br J Cancer (2020) 122(1):94–101. doi: 10.1038/s41416-019-0626-z 31819174 PMC6964696

[38] Nachef M Ali AK Almutairi SM Lee S-H. Targeting SLC1A5 and SLC3A2/SLC7A5 as a potential strategy to strengthen anti-tumor immunity in the tumor microenvironment. Front Immunol (2021) 12:624324. doi: 10.3389/fimmu.2021.624324 33953707 PMC8089370

[39] Dai F Zhang P-B Feng Q Pan X-Y Song S-L Cui J. Cytokine-induced killer cells carrying recombinant oncolytic adenovirus expressing p21Ras scFv inhibited liver Cancer J Cancer (2021) 12(9):2768. doi: 10.7150/jca.51434 33854636 PMC8040716

[40] Du Y Lin X Feng Q Pan X Song S Yang J. Inhibition of human lung cancer cells by anti-p21Ras scFv mediated by the activatable cell-penetrating peptide. Anti-Cancer Drugs (2022) 33(1):e562–72. doi: 10.1097/CAD.0000000000001180 PMC8670359 34338241

[41] Huang C-C Liu F-R Feng Q Pan X-Y Song S-L Yang J-L. RGD4C peptide mediates anti-p21Ras scFv entry into tumor cells and produces an inhibitory effect on the human colon cancer cell line SW480. BMC Cancer (2021) 21(1):1–14. doi: 10.1186/s12885-021-08056-4 33765976 PMC7993510

[42] Qian J Yang M Feng Q Pan X-Y Yang L-L Yang J-L. Inhibition of glioma by adenovirus KGHV500 encoding anti-p21Ras scFv and carried by cytokine-induced killer cells. BMC cancer. (2021) 246(10):1228–38. doi: 10.1177/1535370220986769 PMC8142110 33535808

