# Supplementary figures and images for: ZNF24 regulates the progression of KRAS mutant lung adenocarcinoma by promoting SLC7A5 translation
Source: Front Oncol. 2022 Nov 23;12:1043177. doi: 10.3389/fonc.2022.1043177 (PMC9727282; doi:10.3389/fonc.2022.1043177)

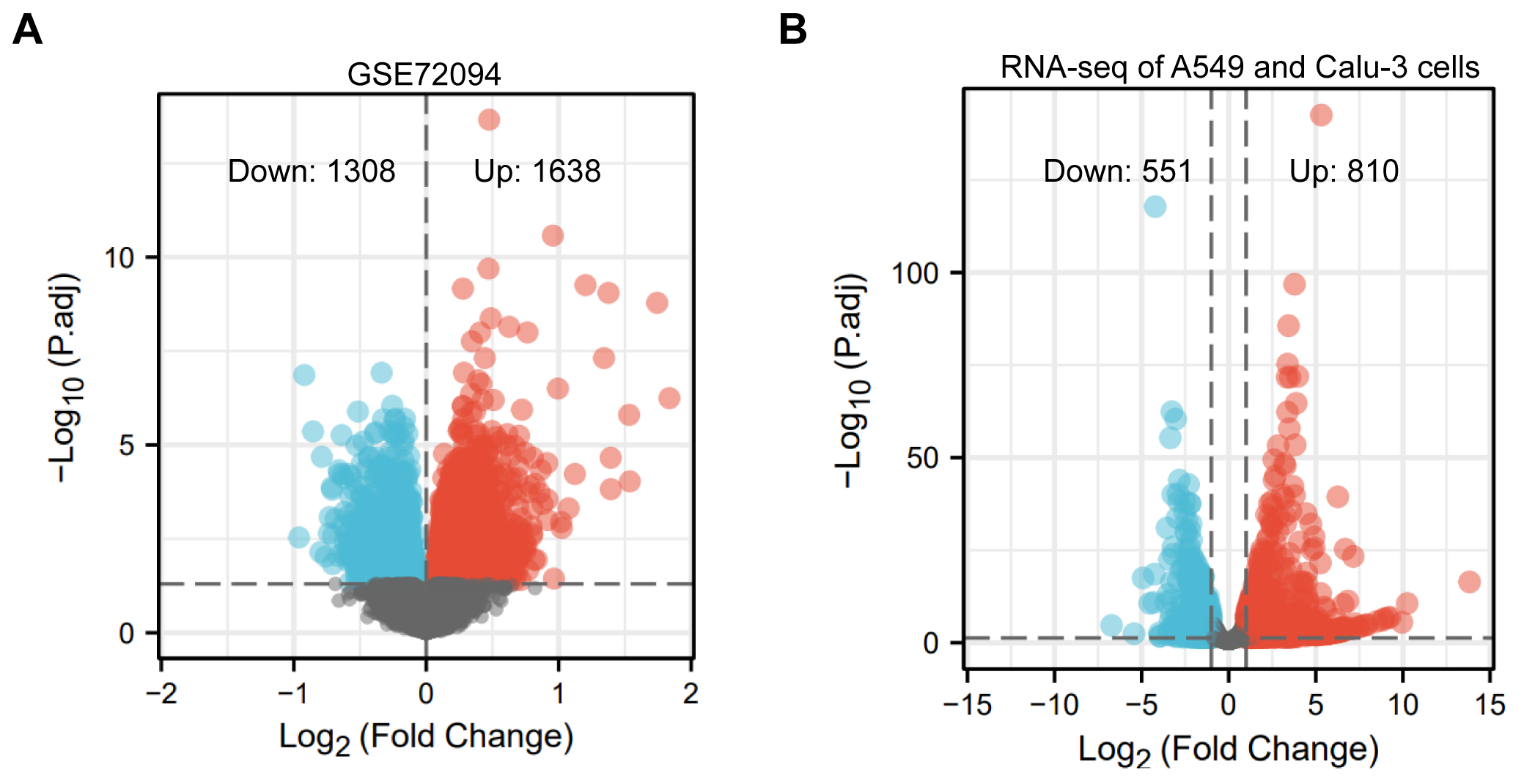

Supplement: Supplementary Figure 1 — After screening for differentially expressed genes, ZNF24 and SLC7A5 were identified as highly expressed genes. (A) Volcano map of differentially expressed genes in the KRAS wild-type groups vs. KRAS mutant groups. (B) Volcano map of differentially expressed genes in shKRAS vs. NC. The threshold of fold change (FC) was set as |log2FC|> 1, P < 0.05. [file Image_1.tif]

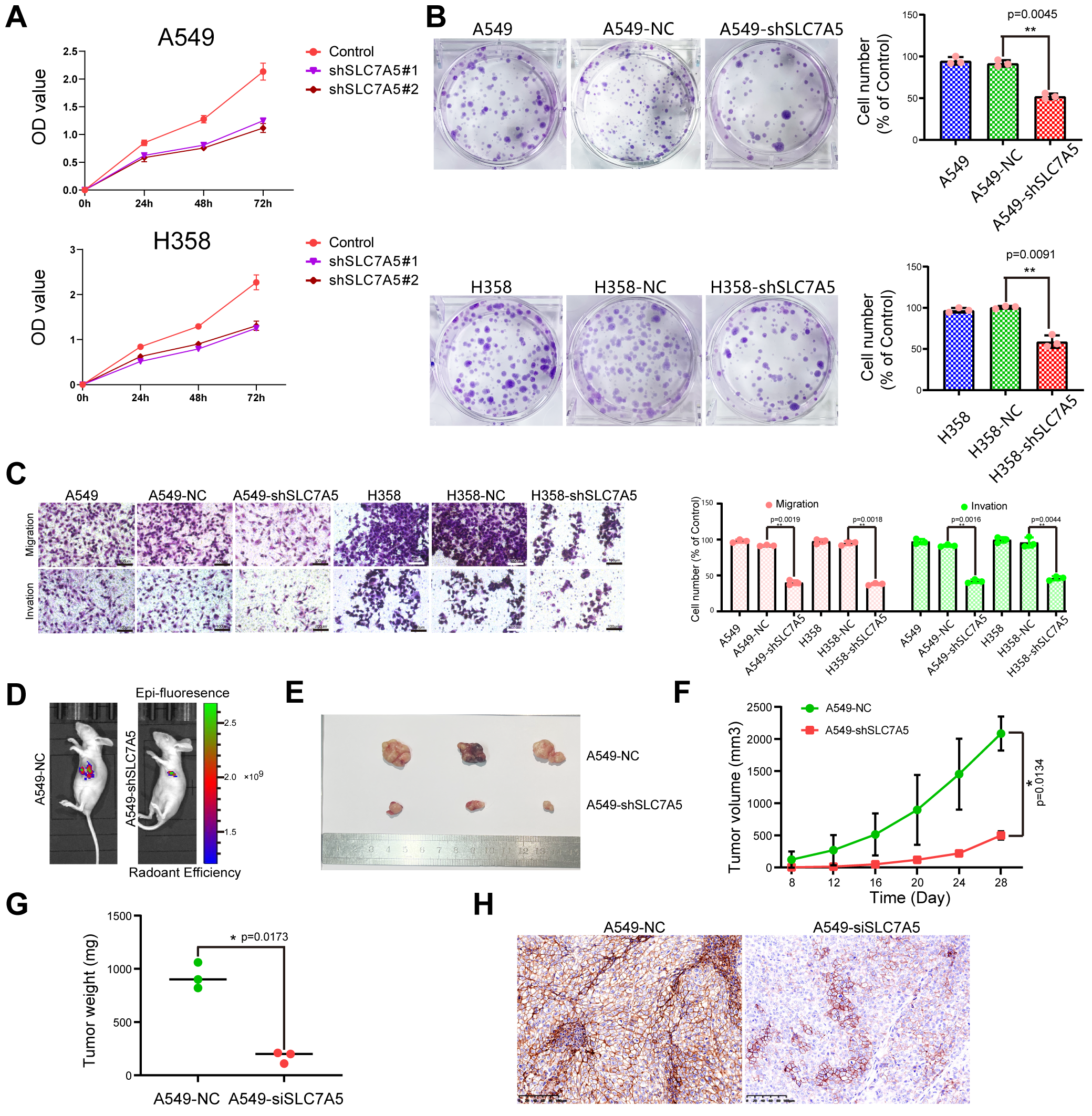

Supplement: Supplementary Figure 2 — Knockdown of SLC7A5 inhibits the growth of KRAS-mutated LUAD cells in vivo and in vitro. (A, B) CCK8 and clone formation assays were performed to detect cell proliferation. (C) Transwell assays were used to measure the invasion ability of cells. (D) Fluorescence imaging of nude mice. (E–G) Measurement of primary tumor volume and weight. (H) Immunohistochemical staining for SLC7A5. shRNA, short hairpin RNA; NC, negative control. Data are shown as means ± SD. P values were calculated with two-tailed Student’s t-test, *p < 0.05, **p < 0.01, ***p < 0.001. [file Image_2.tif]
